# Infant feeding practice and gastrointestinal tolerance: a real-world, multi-country, cross-sectional observational study

**DOI:** 10.1186/s12887-022-03763-8

**Published:** 2022-12-14

**Authors:** Happy Tummy Consortium, M. Y. Jalaludin, S. W. B. Taher, H. B. Kiau, S. B. Hashim, M. B. Yusof, L. W. Khew, M. Juffrie, Saptawati Bardosono, G. Galindez, K. A. I. Waheed, P. Gokhale, M. N. Ibrahim, R. Asghar, H. Shirazi, M. L. M. Perez, D. Kesavelu, A. Edris, A. El Beleidy, M. El Hodhod, M. Elzalabany, H. Hussein, S. Y. Shaaban, A. Elmashad, A. Abdelmoez, O. M. El-Asheer, Y. Vandenplas, Luca Lavalle, Nicolas Sauvageot, Colin Ivano Cercamondi, Delphine Egli, Ivana Jankovic, Yvan Vandenplas

**Affiliations:** 1 Biostatistics & Data, Nestlé Research, Lausanne, Switzerland; 2 Nestlé Product Technology Center – Nutrition, Société Des Produits Nestlé S.A., 1800 Vevey, Switzerland; 3 Vrije Universiteit Brussel, UZ Brussels, KidZ Health Castle, Brussels, Belgium

**Keywords:** Infant formula, Gastrointestinal tolerance, Stooling pattern, Crying time, Infantile colic, Prebiotics, Probiotics

## Abstract

**Background:**

Signs of feeding intolerance, such as gastrointestinal (GI) symptoms, are frequently observed in otherwise healthy formula-fed infants in the first months of life. The primary objective of this observational study was to examine GI tolerance in formula-fed infants (FFI) vs. breastfed infants (BFI) in a real-world setting with a secondary objective being the comparison of infants fed formula with pre- and/or probiotics (FFI_PP) and those fed formula without any pre- and/or probiotics (FFI_noPP) as well as BFI.

**Methods:**

A six-country, cross-sectional study in full-term exclusively/predominantly FFI (*n* = 2036) and BFI (*n* = 760) aged 6–16 weeks was conducted using the validated Infant Gastrointestinal Symptom Questionnaire (IGSQ) and a Feeding Practice and Gut Comfort Questionnaire.

**Results:**

The IGSQ composite score in FFI was non-inferior compared to BFI (mean difference [95%CI]: 0.17 [-0.34, 0.67]; non-inferiority *p*-value < 0.0001) and scores for BFI and FFI were below the threshold of 23, indicating no GI discomfort. Adjusted mean IGSQ scores ± SE were similar in FFI_PP (22.1 ± 0.2) and BFI (22.3 ± 0.3) while FFI_noPP (23.4 ± 0.3) was significantly higher and above 23 indicating some GI discomfort (mean differences [95%CI] FFI_noPP minus FFI_PP and FFI_noPP minus BFI were 1.28 [0.57, 1.98] and 1.09 [0.38, 1.80], respectively; both *p* < 0.01). Hard stools and difficulty in passing stool were more common in FFI compared to BFI (*p* < 0.01) but were less common in FFI_PP compared to FFI_noPP (*p* < 0.01). FFI_PP showed significantly less crying than FFI_noPP and was similar to BFI. Significantly fewer physician-confirmed colic episodes (Rome IV criteria) were reported in FFI_PP compared with FFI_noPP or BFI.

**Conclusions:**

In this real-world observational study, FFI had non-inferior overall GI tolerance compared to BFI. Within FFI, infants receiving formulas with pre- and/or probiotics had a better GI tolerance, improved stooling and less infantile colic compared to those receiving formula without any pre- and/or probiotics and were more similar to BFI.

**Trial registration:**

NCT03703583, 12/10/2018 (https://clinicaltrials.gov/ct2/show/NCT03703583).

**Supplementary Information:**

The online version contains supplementary material available at 10.1186/s12887-022-03763-8.

## Background

In the first months of life, signs of feeding intolerance are often seen in healthy term formula-fed infants (FFI) [1, 2] and may manifest as gastrointestinal (GI) symptoms (such as infrequent stooling or hard stools, spitting-up, or flatulence) and associated behavioral patterns (such as increased fussiness or crying, and dysregulated sleep) [1, 3, 4]. The mechanisms underlying these symptoms and behaviors is thought to be multifaceted but are not yet well understood. Compared to breastfed infants (BFI), depending on the composition of the formula, stools are generally firmer and less frequent in FFI [1–3], likely related to differences in the lipid and mineral fractions of the stools [3]. Colic, flatulence, and regurgitation, while relatively common in all young infants [4, 5], have also been found to be associated with feeding route, with BFI having a lower prevalence of some of these conditions compared to FFI [6, 7]. Parental concern around GI intolerance is high with a large proportion of parents choosing to switch infant formulas for reasons such as regurgitation or vomiting or restlessness behavior [8, 9]. Thus, compositional adjustments of formulas that may beneficially impact symptoms and behaviors related to GI tolerance are important for FFI.

The addition of pre- and/or probiotics to infant formulas has been demonstrated to be effective in improving GI tolerance in randomized controlled trials [10]; however, findings are limited to certain geographic area and were not always consistent. It is therefore important to understand the effectiveness of formula with pre- and/or probiotics in a real-world setting. Therefore, this multi-country study examined GI tolerance in FFI and BFI using the validated Infant Gastrointestinal Symptom Questionnaire (IGSQ) [11] as well as a Feeding Practice and Gut Comfort Questionnaire (FPGCQ) designed for this study. The primary objective was to assess GI tolerance in FFI in comparison with BFI. The secondary objective was to compare a subset of infants fed formula with pre- and/or probiotics (FFI_PP) with those fed formula without any pre- and/or probiotics (FFI_noPP) and with the BFI.

## Methods

### Study design and participants

This multi-country, cross-sectional, observational study was conducted in 6 countries (India, Malaysia, Indonesia, Egypt, Pakistan, and Philippines) from October 2018 to December 2019. For more detailed information about the study centers, locations and institutional review boards, see Supplemental Table 1**,** Additional File 1. Infants were recruited to participate during a routine wellness clinic visit and upon informed consent, study investigators administered two questionnaires to the mothers: IGSQ and FPGCQ. Infants were eligible for the study who were a) healthy, b) born full-term, c) 6–16 weeks of age, and d) either exclusively or predominantly breastfed, or were exclusively or predominantly formula fed. Exclusion criteria included a) premature infants (born < 37 weeks’ gestation), b) infants with parents < 18 years old, c) non-singletons, and d) infants with chronic or acute ongoing illness or food allergies. Each infant’s feeding regimen was assigned according to parent responses to the FPGCQ. Infants with 75% or more of daily feeds for at least the past two weeks coming from breast milk were assigned to BFI, while those with 75% or more of daily feeds for at least the past two weeks from a single formula were assigned to the FFI. Formula composition was used to further characterize the FFI into two subgroups based on whether they were fed a formula containing either prebiotics or probiotics or a combination of both (FFI_PP) or a formula not containing any pre- and/or probiotics (FFI_noPP).

**Table 1:** Demographic, anthropometric and parental characteristics of participating infants, presented as mean, standard deviation (SD), or percentage (%)

Characteristic	BFI (*N* = 760)	FFI (*N* = 2036)	FFI_PP (*N* = 1500)	FFI_noPP (*N* = 501)
Sex				
Male	55.4%	53.2%	52.0%	56.3%
Female	44.6%	46.8%	48.0%	43.7%
Age at enrollment, days	82.1 (24.8)	80.5 (22.6)	80.0 (23.2)	82.5 (24.6)
Delivery mode				
Caesarean	38.4%^d^	41.9%	38.9%	50.9%
Vaginal	61.6%	58.0%	61.0%	49.1%^d^
Unknown	0%	0.1%	0.1%	0%
Gestational age at birth, weeks	38.7 (1.4)	38.6(1.3)	38.6 (1.2)	38.5 (1.3)
Birth weight, g	3068 (468)^a,c^	3011 (496)	3027 (470)	2972 (447)
Birth length, cm	49.3 (2.1)^d^	49.0 (4.5)	49.3 (2.9)	48.6 (1.5)
Birth head circumference, cm	33.5 (1.8)^d^	33.5 (4.5)	33.3 (2.4)	33.9 (1.2)
Weight at visit, g	5503 (965)^a,d^	5302 (948)	5257 (968)	5441 (940)
Length at visit, cm	58.6 (5.5)^b^	58.4 (4.5)	58.3 (3.9)	58.9 (4.5)
Head circumference at visit, cm	39.1 (1.9)	39.0 (1.8)	39.0 (1.9)	39.1 (1.8)
History of gastrointestinal disease in parents				
No	74.5%	75.8%	75.9%	76.8%
Yes	25.5%	24.2%	24.1%	23.2%
Mother’s education				
Low (≤ Primary school)	16.1%	16.2%	15.8%	15.8%
Medium (High school or professional school)	42.0%	46.0%	46.6%	45.5%
High (College +)	42.0%	37.8%	37.6%	38.7%
Father’s education				
Low (≤ Primary school)	12.9%	12.8%	13.3%	10.4%
Medium (High school or professional school)	43.2%	48.8%	49.9%	46.1%
High (College +)	43.9%	38.4%	36.8%	43.5%

BFI, Breastfed infants; FFI, Formula-fed infants; FFI_PP, formula fed infants with prebiotic and/or probiotic; FFI_noPP, formula fed infants with no prebiotic and/or probiotic

^a^ *p* < 0.05 for BFI v All FFI (Chi-square test for categorical variables and t-test for continuous variables)

^b^ *p* < 0.05 for groupwise comparison of BFI, FFI_noPP, and FFI_PP (Chi-square test for categorical variables and t-test for continuous variables)

^c^ *p* < 0.01 for groupwise comparison of BFI, FFI_noPP, and FFI_PP (Chi-square test for categorical variables and t-test for continuous variables)

^d^ *p* < 0.001 for groupwise comparison of BFI, FFI_noPP, and FFI_PP (Chi-square test for categorical variables and t-test for continuous variables)

### Outcome measures

The primary objective of this study was to assess GI tolerance by demonstrating whether IGSQ composite score in FFI is non-inferior to that of BFI. Secondary objectives were to compare the following outcomes between BFI and FFI overall as well as between BFI, and the subgroups FFI_PP and FFI_noPP: IGSQ composite score, GI symptoms and behaviors as measured by the IGSQ individual items as well as stool frequency and consistency, difficulty in passing stool and physician-confirmed colic assessed by FPGCQ.

The IGSQ is a validated questionnaire allowing to calculate a composite score by summing up 13 individual item responses. The composite score ranges from 13–65 with higher scores indicating greater discomfort and internal consistency as measured by the Cronbach’s alpha coefficient was reported to be 0.72 [11]. IGSQ individual item scores were used to assess individual GI symptoms or associated behaviors. The FPGCQ was designed for this study and collected information on feeding practices, colic, and 24-h stool characteristics. Feeding practice measures included counts of breast and formula feeds per day and formula brand where applicable. Information about physician-confirmed infant colic was derived from two questions: “Did the child have colic in the past week?” and “Was your child ever diagnosed with colic?”. If the answer to the second question was yes, follow-up questions for age and feeding regimen at time of diagnosis were asked. Colic was defined according to the ROME IV diagnosis criteria: A) an infant who is < 5 months of age when the symptoms start and stop; B) recurrent and prolonged periods of infant crying, fussing, or irritability reported by caregivers that occur without obvious cause and cannot be prevented or resolved by caregivers and C) no evidence of infant failure to thrive, fever, or illness. Study investigators explained the colic definition to the mothers when asking the questions. Stool frequency, consistency, and difficulty passing stool for each stool in the past 24 h were recorded, with stool consistency rated on a validated 4-point scale (1-watery, 2-loose, 3-formed, 4-hard) [12].

Demographic characteristics including age at enrollment, sex, gestational age, delivery type, mother’s and father’s education level (low = primary school or lower; medium = high school or professional school; high = college or higher) and history of gastrointestinal disease in parents were recorded as well as infant weight, height and head circumference at birth. Infant weight, height, and head circumference were measured at the time when the two questionnaires were administered.

### Statistical analysis

Statistical power for the primary analysis was based on data from a prior study [11] assuming an IGSQ score of 18 for BFI, a non-inferiority margin of 2.7 (15% of the BFI value), a standard deviation of 6.0, a one-sided 97.5% confidence interval, and a statistical power greater than 90%. With the primary objective being the demonstration of non-inferior IGSQ composite score in FFI versus BFI with a more or less equal distribution of infants among the study countries, sample size calculation indicated that 120 infants per group (BFI and FFI) would be required. Considering the potential heterogeneity of formula compositions in FFI and in order to have enough statistical power for the subgroup analysis of FFI_PP and FFI_noPP, approximately 350–500 subjects (n ~ 120 for BFI and n ~ 230–380 for FFI) were included in each country.

Descriptive statistics, including means, standard deviations (SD) for continuous variables and percentages for categorical variables were reported. For the primary objective, non-inferiority of the IGSQ composite score in FFI compared with BFI was based on the upper bound of the two-sided 95% confidence interval of the analysis of covariance (ANCOVA) model which had to be lower than the non-inferiority margin which was defined to be 15% of the average IGSQ composite score of BFI in our study (= 3.35). The student’s t-test and the chi-square test were used to compare demographic characteristics between feeding regimens. Continuous outcomes, including the IGSQ composite score, IGSQ individual item scores, and the FPGCQ stool consistency score, were analyzed using ANCOVA, while binary outcomes, including difficulty passing stool, and physician-confirmed colic, were modeled using logistic regression. The FPGCQ stool frequency was modeled using a negative binomial model. All statistical models including feeding regimen (BFI/FFI or BFI/FFI_noPP/FFI_PP) were further adjusted for study site, infant age, sex, delivery type, history of GI disease in parents, and mother’s education. Tests were two-sided with an alpha level equal to 5%. Analyses were conducted using SAS/STAT software version 9.3 or higher (SAS Institute Inc., Cary, NC, USA).

## Results

In this study, 2796 infants were enrolled (760 in BFI and 2036 in FFI) with 1500 in FFI_PP and 501 in FFI_noPP. Thirty-five infants were not categorized into FFI_PP or FFI_noPP due to unknown formula composition. Demographics and anthropometrics are reported in Table 1 for BFI and FFI overall and also for FFI_PP and FFI_noPP. Vaginal births were similar between the BFI (61.6%) and FFI (58.0%), while the FFI_noPP had a lower prevalence of vaginal birth (49.1%) compared with FFI_PP (61.0%) or BFI (61.6%) (*p* < 0.001). Birth weight was statistically greater in BFI compared with FFI (*p* = 0.005) with a difference that by the investigators was deemed to be of minimal clinical relevance; birth length and head circumference did not vary between the BFI and FFI. When comparing the BFI, FFI_PP, and FFI_noPP, birth weight and birth length were higher in the BFI and FFI_PP compared with FFI_noPP (*p* = 0.005 and < 0.001, respectively) while head circumference at birth was lower in BFI and FFI_PP compared with FFI_noPP (*p* < 0.001) with differences that by the investigators were deemed to be of minimal clinical importance. At the study visit, weight and length were statistically significantly different in BFI compared with the formula-fed groups; no differences were seen at the study visit for head circumference.

**Fig. 1:**
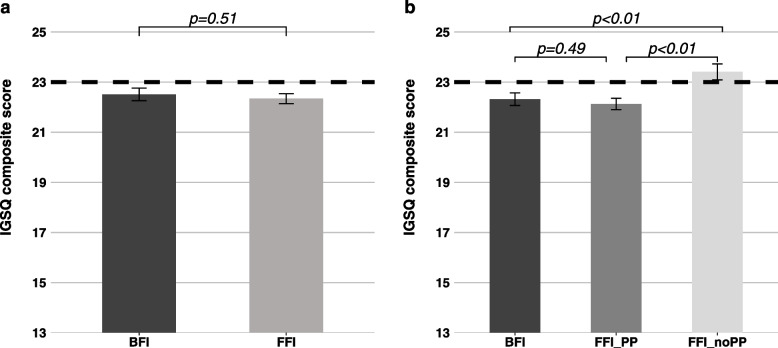
Adjusted mean IGSQ composite scores with SE as whiskers by feeding regimens.**.** Group comparison done by analysis of covariance adjusted for feeding regimen, study site, infant age, sex, delivery type, history of GI disease in parents, and mother’s education. IGSQ composite score can range from 13–65, with higher values indicating greater discomfort. Dotted line marks threshold of 23 indicating certain GI discomfort (> 23 to 30; > 30 to 65 indicates GI distress) and essentially no GI issues (≤ 23). BFI, Breastfed infants; FFI, Formula-fed infants; FFI_PP, formula fed infants with prebiotic and/or probiotic; FFI_noPP, formula fed infants with no prebiotic and/or probiotic; GI, gastrointestinal; IGSQ, Infant Gastrointestinal Symptom Questionnaire. *n* = 760 in BFI; *n* = 2036 in FFI; *n* = 1500 in FFI_PP; *n* = 501 in FFI_noPP. FFI_PP and FFI_noPP are subgroups of FFI. Thirty-five infants were not categorized into FFI_PP or FFI_noPP because of unknown formula composition

The adjusted mean IGSQ composite scores for the BFI and FFI are shown in Fig. 1a; both mean scores were below the IGSQ threshold of 23 commonly used to indicate GI discomfort [11]. Non-inferiority of the FFI compared to the BFI was demonstrated with the upper bound of the 95% CI of the difference of the adjusted means (FFI minus BFI) being less than the non-inferiority margin of 3.35 (mean difference [95%CI]: 0.17 [-0.34, 0.67]; non-inferiority *p*-value < 0.0001). Figure 1b shows the IGSQ composite scores ± SE for BFI (22.3 ± 0.3), FFI_PP (22.1 ± 0.2), and FFI_noPP (23.4 ± 0.3). IGSQ composite score in FFI_PP was lower than in FFI_noPP indicating better GI tolerance in FFI_PP than in FFI_noPP (mean differences FFI_noPP minus FFI_PP [95%CI]: 1.28 [0.57, 1.98]; *p* < 0.01). No significant difference was observed between FFI_PP and BFI (mean difference FFI_PP minus BFI [95%CI]: -0.19 [-0.73, 0.35]; *p* = 0.49) indicating no difference in GI symptoms between BFI and FFI_PP and both groups had score below the threshold of 23. In contrast, IGSQ composite score in FFI_noPP was above the 23-cut-off indicating some GI discomfort and was also higher than in BFI (mean differences FFI_noPP minus BFI [95%CI]: 1.09 [0.38, 1.80]; *p* < 0.01).

Comparisons of the individual IGSQ item scores are shown in Table 2**.** BFI had less hard stools (*p* < 0.01) and less difficulty in passing stool (*p* < 0.01) than FFI or the subgroups FFI_PP and FFI_noPP (both *p* < 0.05). Compared to FFI_noPP, FFI_PP had less hard stools (*p* = 0.01) and tended to have less difficulty in passing stool (*p* = 0.05). FFI_PP had significantly lower crying time, lower frequency of spit up, less volume of milk spit up, less discomfort when spitting-up and less discomfort due to gas than FFI_noPP and was similar to BFI whose scores were also lower for these items compared with FFI_noPP (all *p* < 0.05; except for frequency of spit up for which *p* = 0.06). FFI and the subgroup FFI_PP had significantly better scores for the ability to soothe crying than BFI, and FFI_PP had significantly less crying after feeding compared to BFI. There were no significant differences between the feeding groups for frequency of arching back, fussiness, or flatulence and ability to sooth fussiness.

**Table 2:** Adjusted mean difference in individual IGSQ item scores by feeding regimen^1^

IGSQ Domain	IGSQ Item	Comparison	Mean Difference	95% CI	*p*-value
Stool	1. Hard stools: *“How many times did your baby pass a hard stool?”*	FFI—BFI	0.10	0.04, 0.15	< 0.01
		FFI_noPP—FFI_PP	0.09	0.02, 0.16	0.01
		FFI_PP—BFI	0.06	0.01, 0.12	0.03
		FFI_noPP—BFI	0.16	0.08, 0.23	< 0.01
	2. Difficulty in passing stool: *“How many times did your baby have difficulty when passing a bowel movement?”*	FFI—BFI	0.12	0.05, 0.19	< 0.01
		FFI_noPP—FFI_PP	0.10	0.00, 0.19	0.05
		FFI_PP—BFI	0.09	0.01, 0.16	0.02
		FFI_noPP—BFI	0.18	0.09, 0.28	< 0.01
Spitting-up/Vomiting	3. Frequency of spit up: *“How many times did milk come out of your baby’s mouth?”*	FFI—BFI	0.01	-0.08, 0.10	0.83
		FFI_noPP—FFI_PP	0.16	0.03, 0.28	0.01
		FFI_PP - BFI	-0.03	-0.13, 0.06	0.50
		FFI_noPP—BFI	0.12	0.00,0.25	0.06
	4. Volume of milk spit up: *“How much milk usually came out each time?”*	FFI—BFI	-0.02	-0.09, 0.05	0.52
		FFI_noPP—FFI_PP	0.18	0.08, 0.27	< 0.01
		FFI_PP—BFI	-0.07	-0.14, 0.00	0.05
		FFI_noPP—BFI	0.11	0.01, 0.20	0.03
	5. Discomfort when spitting up: *“How often did your baby seem uncomfortable or fussy when milk came out of his or her mouth?”*	FFI—BFI	0.03	-0.04, 0.10	0.41
		FFI_noPP- FFI_PP	0.14	0.04, 0.24	< 0.01
		FFI_PP—BFI	-0.01	-0.09, 0.06	0.76
		FFI_noPP—BFI	0.13	0.03, 0.23	0.01
	6.Frequency of arching back: *“How many times did your baby arch his or her back as if in pain when milk came out of his or her mouth?”*	FFI—BFI	0.00	-0.06, 0.05	0.96
		FFI_noPP—FFI_PP	0.03	-0.05, 0.11	0.44
		FFI_PP—BFI	-0.01	-0.07, 0.05	0.80
		FFI_noPP—BFI	0.02	-0.06, 0.10	0.57
Crying	7. Total crying time: *“How much total time did your baby usually cry in a day?”*	FFI—BFI	0.07	-0.004, 0.14	0.06
		FFI_noPP—FFI_PP	0.19	0.09, 0.29	< 0.01
		FFI_PP—BFI	0.01	-0.06, 0.09	0.71
		FFI_noPP—BFI	0.21	0.10, 0.31	< 0.01
	8. Unable to soothe crying: *“How many times were you unable to soothe your baby to stop his or her crying?”*	FFI—BFI	-0.11	-0.18, -0.03	< 0.01
		FFI_noPP—FFI_PP	0.06	-0.05, 0.17	0.26
		FFI_PP—BFI	-0.12	-0.20, -0.04	< 0.01
		FFI_noPP—BFI	-0.06	-0.17, 0.05	0.26
	9. Crying after feeding: *“How many times did your baby cry during or right after a feeding because the milk bothered your baby?”*	FFI—BFI	-0.07	-0.14, 0.01	0.07
		FFI_noPP—FFI_PP	0.08	-0.02, 0.19	0.11
		FFI_PP—BFI	-0.09	-0.17, -0.01	0.03
		FFI_noPP—BFI	-0.01	-0.11, 0.10	0.92
Fussiness	10. Frequency of fussiness: *“On how many days was your baby fussy?”*	FFI – BFI	-0.02	-0.11, 0.06	0.56
		FFI_noPP—FFI_PP	0.02	-0.09, 0.14	0.72
		FFI_PP—BFI	-0.02	-0.11, 0.07	0.62
		FFI_noPP—BFI	0.00	-0.12, 0.12	0.98
	11. Unable to soothe fussiness: *“How many times were you unable to soothe your baby when he or she was fussy?”*	FFI – BFI	-0.05	-0.12, 0.02	0.18
		FFI_noPP—FFI_PP	0.06	-0.04, 0.15	0.24
		FFI_PP—BFI	-0.06	-0.13, 0.02	0.12
		FFI_noPP—BFI	0.00	-0.10, 0.10	0.98
Flatulence	12. Frequency of gassiness: *“How many times in a usual day was your baby gassy?”*	FFI – BFI	0.08	-0.01, 0.17	0.09
		FFI_noPP—FFI_PP	0.06	-0.07, 0.18	0.38
		FFI_PP—BFI	0.06	-0.03, 0.16	0.20
		FFI_noPP—BFI	0.12	-0.01, 0.25	0.07
	13. Discomfort due to gas: *“How often did gas seem to make your baby uncomfortable or fussy?”*	FFI—BFI	0.02	-0.06, 0.09	0.66
		FFI_noPP—FFI_PP	0.14	0.03, 0.25	0.01
		FFI_PP—BFI	-0.02	-0.11, 0.06	0.57
		FFI_noPP—BFI	0.11	0.004, 0.22	0.04

BFI, Breastfed infants; CI, Confidence Interval; FFI, Formula-fed infants; FFI_PP, formula fed infants with prebiotic and/or probiotic; FFI_noPP, formula fed infants with no prebiotic and/or probiotic; IGSQ, Infant Gastrointestinal Symptom Questionnaire. *n* = 760 in BFI; *n* = 2036 in FFI; *n* = 1500 in FFI_PP; *n* = 501 in FFI_noPP. FFI_PP and FFI_noPP are subgroups of FFI. Thirty-five infants were not categorized into FFI_PP or FFI_noPP because of unknown formula composition. P-values computed from ANCOVA models including feeding practice as the independent variable and infant sex, age, study center, delivery type, mother’s education and diagnosed functional gastrointestinal disorder in the parents as potential confounders.^1^ Higher scores indicate greater discomfort

**Fig. 2:**
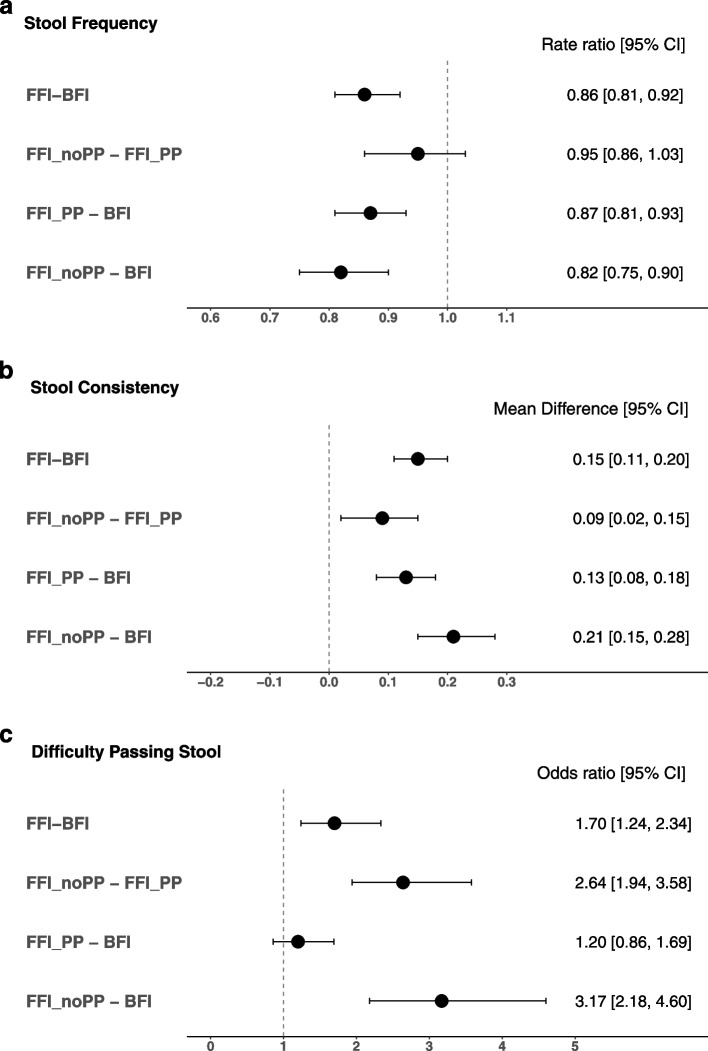
Comparisons of stool characteristics between feeding groups. Comparisons of stool characteristics between the feeding groups are shown as rate ratios for stool frequency (**2a**), mean differences for stool consistency (**2b**), and odds ratios for difficulty passing stool (**2c**). The vertical line shows the reference value for each measure. Stool frequency and consistency and difficulty in passing stool were measured using the Feeding Practice and Gut Comfort Questionnaire, which collected number of stool and consistency for each stool in the 24 h prior to the administration of the questionnaire, as well as whether the infant had difficulty passing each stool. Stool consistency was measured using a 4-point scale (1 = Watery, 2 = Loose, 3= Formed, 4 = Hard). Stool frequency was modeled using negative binomial regression, consistency was modeled using ANCOVA, and difficulty passing stool was modeled using logistic regression. In addition to feeding regimen, models were further adjusted for study site, infant age, sex, delivery type, history of GI disease in parents, and mother’s education. BFI, Breastfed infants; CI, Confidence Interval; FFI, Formula-fed infants; FFI_PP, formula fed infants with prebiotic and/or probiotic; FFI_noPP, formula fed infants with no prebiotic and/or probiotic. *n* = 760 in BFI; *n* = 2036 in FFI; *n* = 1500 in FFI_PP; *n* = 501 in FFI_noPP. FFI_PP and FFI_noPP are subgroups of FFI. Thirty-five infants were not categorized into FFI_PP or FFI_noPP because of unknown formula composition

The mean (standard error [SE]) stool frequency in the past 24-h period was 2.0 (0.06) in BFI, 1.7 (0.03) in FFI, 1.7 in FFI_PP and 1.8 (0.1) in FFI_noPP with corresponding rate ratios indicating significantly lower stool frequency in FFI as well as FFI_PP and FFI_noPP compared to BFI (all *p* < 0.01) (Fig. 2a). FFI had significantly harder stool consistency compared to BFI (adjusted mean [SE] stool consistency score: 2.07 [0.02] vs 1.92 [0.02]; *p* < 0.01) (Fig. 2b). The stool consistency score for FFI_PP (adjusted mean [SE]: 2.04 [0.02]) indicated significantly softer stool than for FFI_noPP (adjusted mean [SE]: 2.12 $[$0.03]; *p* < 0.01) with both subgroups having significantly harder stool consistency than BFI (*p* < 0.01) (Fig. 2b). FFI were more likely to have difficulty passing stool than BFI based on the FPGCQ (*p* < 0.01; Fig. 2c)**.** This result confirmed the IGSQ result on the same outcome and was likely driven by FFI_noPP which were more likely to have difficulty passing stool compared to both BFI (*p* < 0.0001) and FFI_PP (*p* < 0.0001), while there was no difference between BFI and FFI_PP.

**Fig. 3:**
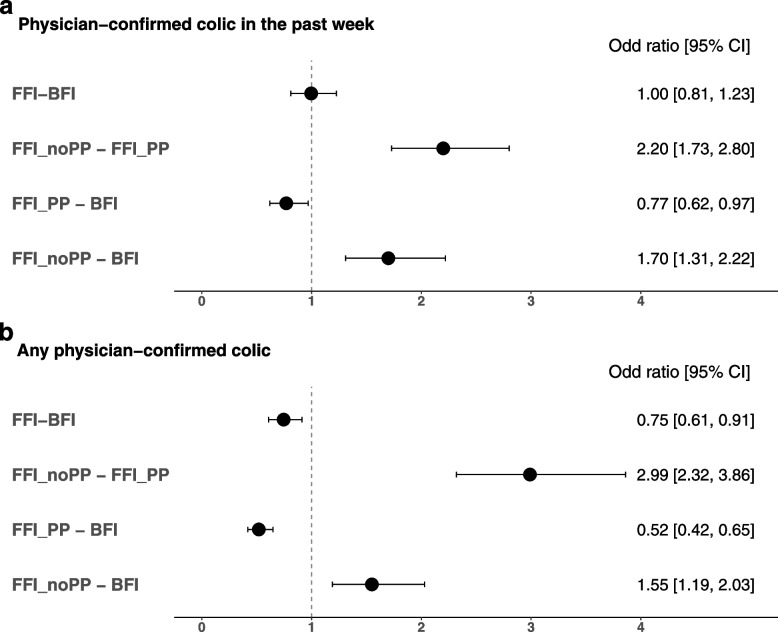
Odds ratios with 95% confidence intervals comparing physician-confirmed colic between feeding groups. The vertical line shows reference value for the odds ratios. Physician-confirmed colic were measured using the Feeding Practice and Gut Comfort Questionnaire (“Was your child ever diagnosed colic?” and “Did the child have colic in the past week?”). Both outcomes were modeled using logistic regression and adjusted for study site, infant age, sex, delivery type, history of GI disease in parents, and mother’s education. BFI, Breastfed infants; CI, Confidence Interval; FFI, Formula-fed infants; FFI_PP, formula fed infants with prebiotic and/or probiotic; FFI_noPP, formula fed infants with no prebiotic and/or probiotic. *n* = 760 in BFI; *n* = 2036 in FFI; *n* = 1500 in FFI_PP; *n* = 501 in FFI_noPP. FFI_PP and FFI_noPP are subgroups of FFI. Thirty-five infants were not categorized into FFI_PP or FFI_noPP because of unknown formula composition

Physician-confirmed colic in the past week was observed in 23.6% of BFI, 23.5% of FFI, 19.3% of FFI_PP and 33.8% of FFI_noPP. Adjusted odds ratios (ORs) for physician-confirmed colic were not different between BFI and FFI (*p* = 0.98; Fig. 3). However, odds were greater for FFI_noPP compared to both FFI_PP (OR = 2.20, 95%CI: 1.73, 2.80; *p* < 0.0001) and BFI (OR = 1.70, 95%CI: 1.31, 2.22; *p* < 0.0001) indicating more colic in FFI_noPP. Lower odds for FFI_PP compared to BFI were also observed (OR = 0.77, 95%CI: 0.62, 0.97; p = 0.02). Any physician-confirmed colic in the past occurred in 28.4% of BFI, 23.0% of FFI, 17.3% of FFI_PP, and 36.9% of FFI_noPP. Odds were lower in FFI compared to BFI (OR = 0.75, 95% CI: 0.61, 0.91; *p* = 0.0045). FFI_PP had significantly lower odds for any colic in the past than BFI while FFI_noPP had significantly greater odds compared to both BFI and FFI_PP (Fig. 3).

## Discussion

This multi-country, cross-sectional observational study using the validated IGSQ was conducted to examine real-world GI tolerance of different feeding practices in infants. We demonstrated non-inferiority of the IGSQ composite score in FFI infants compared to those who were breastfed. Importantly, when the type of formula was considered, we found that overall GI tolerance among infants fed formula containing pre and/or probiotics was significantly better than in infants receiving a formula without any pre- and probiotics and comparable to BFI. The IGSQ composite score for BFI and FFI_PP was below 23 indicating no GI distress in these infants, while for FFI_noPP, it was above 23 commonly used to denote certain GI distress. The good GI tolerance in FFI_PP likely drove the non-inferiority observed for FFI vs. BFI, offsetting the impaired GI tolerance in FFI_noPP. The mechanisms on how pre- or probiotics impact GI tolerance are not yet fully understood. A balanced gut microbiota has been noted to be one of the major factors promoting proper gut function and development; hence likely contributes to GI tolerance [13].This balance can be mediated by prebiotics and probiotics which have been shown to have a multifaceted, beneficial influence on the gut microbiota [10, 14, 15]. Prebiotics have also been demonstrated to affect stooling via their fermentation by GI bacteria which leads to augmented microbial mass and then increased fecal water content, resulting in softer stools. Additionally, the fermentation of prebiotics which results in selective growth of lactobacillus and bifidobacteria species helps produce short-chain fatty acids which may also increase the water content of the fecal mass and stimulate gastrointestinal motility which then facilitates stooling [16].

The IGSQ scores in our study are in line with those observed in previous studies on infant formulas supplemented with pre and/or probiotics. In an RCT of a test formula containing *Bifidobacterium (B.) lactis* and 2’FL compared to a control formula with *B. Lactis* alone, no IGSQ differences were shown between the two groups (20.9 for test vs. 20.7 for control) [17]. Similarly, an interventional real-world evidence study in Spain found no difference in IGSQ scores after 8 weeks among infants receiving a formula with 2’fucosyllactose and lacto-n-neotetraose, infants receiving a mixture of formula and breastmilk, and exclusive BFI (mean IGSQ scores 20.7, 20.4, and 19.9, respectively) [18]. Another real-world study in China had similar IGSQ scores among infants receiving a formula with added sn-2 palmitate and oligofructose (sn-2 + OF), a mixture of sn-2 + OF formula and breastmilk, and breastmilk alone (mean IGSQ scores after 18 days were 17.5, 18.2, and 16.3, respectively) [19]. Collectively, these results, including the present study, demonstrate good GI tolerance for pre- and/or probiotic supplemented formulas comparable to that in BFI. The differences in the absolute mean IGSQ composite scores are likely due to different geographical regions where the studies were conducted and related to the parental perception of GI tolerance as well as cultural differences or differences in parental health literacy around feeding practices across countries and regions, all of which may impact infant GI tolerance.

Stool consistency has also been examined in several studies of pre and/or probiotic-supplemented formulas. In our study, we observed that the infants receiving formula without any pre- and probiotics had harder stools and more difficulty of passing stool compared to both the BFI and the infants receiving formula with pre and/or probiotics. A review of studies that evaluated stool characteristics among infants receiving a mixture of prebiotics found softer stools in the infants receiving formula with prebiotics in 4 out of 5 studies [16]. Multiple other studies have also demonstrated that infant formulas supplemented with prebiotics or mixtures of pre- and probiotics are associated with softer stools [17, 20–23]. Notably the previous studies are all randomized clinical trials; thus, it is reassuring that similar effects for stooling were observed in our real-world observational study.

Our results add to the growing body of literature demonstrating potential benefits of pre- and probiotics on colic. In our study, we observed higher odds for colic in the infants receiving formula with no pre and/or probiotics compared to the BFI and the infants receiving formula with pre and/or probiotics. Several clinical studies have examined probiotics in relation to colic or behavioral patterns associated with colic. A recent systemic review including 15 RCTs and 5 meta-analyses found that the quality of the body of supporting the administration of probiotics to breastfed infants to relieve colic symptoms was level A. This was on ≥ 50% reduction in crying time compared with placebo. The quality of the body of evidence supporting the administration of probiotics to relieve colic symptoms in formula-fed infants was level B due to the limited numbers of formula-fed infants in the included studies [24]. With regards to colic prevention, mixed evidence has been reported for probiotics. In one RCT, FFI receiving probiotics had less crying time than the control group [25] and in another RCT, BFI and FFI had significantly fewer colic symptoms at 3 months than the control group [26]. On the other hand, in two clinical trials, there were no significant differences in the development of colic symptoms between BFI or FFI receiving probiotics and the control group [27, 28]. Regarding prebiotics, data about colic management and prevention is limited to a few RCTs. A trial in Italy reported that in a group receiving formula supplemented with galacto-oligosaccharides, the incidence of colic was lower than in the control group [29]. Furthermore, colicky infants consuming formula with fructo- and galacto-oligosaccharides had reduced crying episodes compared to their control peers [30] and non-colicky infants consuming a similar formula had lower overall crying time and less infantile colic episodes than the control group [31]. Overall, these findings on pre—or probiotics are consistent with ours. In agreement with the literature, we found a relatively high prevalence of colic in BFI. Having this high prevalence of colic in BFI, likely drove the difference in colic we found between BFI and FFI_PP.

A major strength of this study is the robust sample size including data from six different countries. These were either lower or upper middle-income countries for which data on GI tolerance in FFI was limited and to our knowledge, our study is the first real-world evidence study covering such a broad spectrum of different infant formulas in a large population. Additionally, the use of the IGSQ is a strength as it provides a standardized metric for GI tolerance that can be utilized by clinicians and researchers across studies. One limitation of this study is that it was cross-sectional in design. However, the IGSQ covered a period of one week and in the FPGCQ, we retrospectively assessed colic prevalence over a longer period. An additional limitation is that infants were fed many different formulas not only differing in pre- and/or probiotics; thus, controlling for all factors potentially impacting GI tolerance was out of the scope.

## Conclusions

In this real-world observational study, infant formulas containing pre and/or probiotics were associated with overall improved digestive tolerance, providing supportive data of previously established efficacy of such formulas in randomized controlled trials. The results of this real-world study are very similar to those seen in randomized controlled trials which is a reassurance to caregivers as well as clinicians that the effects of formula supplemented with pre and/or probiotics seen in more tightly controlled trial settings are likely generalizable to a broader real-world population.

## Supplementary Information

**Additional file 1.**

## Data Availability

The datasets used and/or analysed during the current study are available from the corresponding author on reasonable request.

## Abbreviations

GI: Gastrointestinal
FFI: Formula-fed infants
BFI: Breastfed infants
FFI_PP: Infants fed formula with pre- and/or probiotics
FFI_noPP: Infants fed formula without any pre- and/or probiotics
IGSQ: Infant Gastrointestinal Symptom Questionnaire
FPGCQ: Feeding Practice and Gut Comfort Questionnaire
