# Supplementary material for: Infant feeding practice and gastrointestinal tolerance: a real-world, multi-country, cross-sectional observational study
Source: BMC Pediatr. 2022 Dec 14;22:714. doi: 10.1186/s12887-022-03763-8 (PMC9749164; doi:10.1186/s12887-022-03763-8)
Supplement: Supplementary file 1 — Additional file 1. [file 12887_2022_3763_MOESM1_ESM.pdf]

Additional File 1

Supplemental table 1. Study Centers and Institutional Review Board Approvals

Center	Location	Approving Institutional Review Board/Ethics Committee
Ain Shams University	Cairo, Egypt	Ain Shams University, Faculty of Medicine, Research Ethics Committee<br>FWA 00017585 / FMASU P93/2018
Cairo University	Cairo, Egypt	Cairo University, Faculty of Medicine, Research Ethics Committee<br>REF: N-106-2018
Alexandria University	Alexandria, Egypt	Alexandria University, Faculty of Medicine, Ethics Committee,<br>IRB #: 0000-7555
Zagazig University	Zagazig, Egypt	Zagazig University, Faculty of Medicine, Institutional Review Board<br>IRB #: 4872
Tanta University	Tanta, Egypt	Tanta University, Faculty of Medicine, Research Ethics Committee Quality Assurance Office<br>REF: 32541/09/18
Assiut University	Assiut, Egypt	Assiut University, Faculty of Medicine, Institutional Review Board<br>IRB #: 17400010
The Children Hospital & Institute of Child Health	Lahore, Pakistan	The Children Hospital, Lahore, Institutional Review Board<br>IERB #: 02/2019
Karachi Medical & Dental College	Karachi, Pakistan	National Bioethics Committee (NBC) Pakistan<br>REF: No.4-87/NBC-385/19/924
National Institute of Child Health	Karachi, Pakistan	National Institute of Child Health, Institutional Ethical Review Board (IERB)<br>IERB #: 02/2019
Benazir Bhutto Hospital	Rawalpindi, Pakistan	Rawalpindi Medical University, Institutional Research Forum<br>REF#: R-12/RM-1
Children's Hospital, Pakistan Institute of Medical Sciences	Islamabad, Pakistan	Children Hospital, Pakistan Institute of Medical Sciences G-8/3,<br>REF #: 390

Quezon City City Health Office<br>Cebu City Health Office<br>Davao City Health Office	Quezon City, Philippines<br>Cebu City, Philippines<br>Davao del Sur, Philippines	St. Luke's Medical Center College of Medicine, Research Ethics Committee<br>RP 18-12
Bantul Hospital<br>Kulonprogo hospital<br>Sleman hospital<br>Community Health Center Cilincing<br>Community Health Center Grogol	Yogyakarta, Indonesia<br>Yogyakarta, Indonesia<br>Yogyakarta, Indonesia<br>Jakarta Utara, Indonesia<br>Jakarta Barat, Indonesia	Medical and Health Research Ethics (MHREC)<br>Faculty of Medicine Gadjah Mada University<br>REF: KE/FK/0480/EC/2018
Klinik Kesihatan Simpang Kuala<br>Klinik Kesihatan Bandar Botanik	Alor Setar, Malaysia<br>Klang, Malaysia	University of Malaya Medical Research Ethics Committee<br>MREC ID NO: 201892-6636
Klinik Kesihatan Endau<br>Klinik Kesihatan Bandar<br>Klinik Kesihatan Luyang	Mersing, Malaysia<br>Kota Bharu, Malaysia<br>Kota Kinabalu, Malaysia	Ministry of Health Malaysia - Medical Research and Ethics Committee<br>REF: KKM/NIHSEC/P18-1756
Childrens Clinic and Vaccination Centre<br>Hiranandani Hospital	Maharashtra, India<br>Mumbai, India	Dr L H Hiranandani Hospital, Institutional Ethics Committee<br>REF: IECM/Res./October-2018/0001
ICH Hospital	Kolkata, India	Institute of Child Health, Institutional Ethics Committee<br>REF: IEC/168/2018
Apollo Children's Hospital	Chennai, India	Apollo Hospital, Institutional Ethics Committee - Clinical Studies<br>REF: IEC-CS App. No.: ACH-005/08-18
